# Steady-state running rate sets the speed and accuracy of accumulation of swimming bacteria

**DOI:** 10.1016/j.bpj.2022.08.012

**Published:** 2022-08-31

**Authors:** Margaritis Voliotis, Jerko Rosko, Teuta Pilizota, Tanniemola B. Liverpool

**Affiliations:** 1 College of Engineering, Mathematics and Physical Sciences, University of Exeter, Exeter, United Kingdom; 2 School of Life Sciences, University of Warwick, Coventry, United Kingdom; 3 Centre for Synthetic and Systems Biology, University of Edinburgh, Edinburgh, United Kingdom; 4 School of Mathematics, University of Bristol, Bristol, United Kingdom; 5 BrisSynBio, Life Sciences Building, University of Bristol, Bristol, United Kingdom

## Abstract

We study the chemotaxis of a population of genetically identical swimming bacteria undergoing run and tumble dynamics driven by stochastic switching between clockwise and counterclockwise rotation of the flagellar rotary system, where the steady-state rate of the switching changes in different environments. Understanding chemotaxis quantitatively requires that one links the measured steady-state switching rates of the rotary system, as well as the directional changes of individual swimming bacteria in a gradient of chemoattractant/repellant, to the efficiency of a population of bacteria in moving up/down the gradient. Here we achieve this by using a probabilistic model, parametrized with our experimental data, and show that the response of a population to the gradient is complex. We find the changes to the steady-state switching rate in the absence of gradients affect the average speed of the swimming bacterial population response as well as the width of the distribution. Both must be taken into account when optimizing the overall response of the population in complex environments.

## Significance

In nature, bacteria live in complex environments such as the mammalian gastrointestinal tract or the soil. Understanding how bacteria achieve robust navigation in these environments while growing, and when multiple competing stimuli are present, captures interest from a variety of disciplines, including biology, medicine, physics, and bio-inspired design in engineering. Here, motivated by experimental findings showing that environmental conditions affect the effective diffusion constant of swimming bacteria, we develop a probabilistic model of chemotaxis and use it to study how such changes can affect the ability of bacteria to quickly and accurately find their targets.

## Introduction

Bacterial self-propulsion, in particular flagellated motility, is a phenomenon that captures interest from a variety of disciplines, ranging from physics (1), biology, and medicine (2) to bio-inspired design in engineering (3). Interest in motility is often in the context of chemotaxis, in which membrane-bound proteins acting as chemo-receptors sense the presence of certain chemicals in the environment and affect the flagellar rotation in order to move toward or away from the source (4). Much of the research on chemotaxis focuses on dilute aqueous media with or without a single chemical gradient (4,5). This reductionist approach has been invaluable and generated a large body of knowledge about the underlying mechanisms. Briefly, and taking the example of the model organism *Escherichia coli*, the bacterium swims by rotating a flagellar filament bundle that propels its body through the environment (6,7). Each flagellum consists of a long, thin, helical filament attached to a bacterial flagellar motor (BFM), which drives its rotation at rates exceeding 100 Hz. It spins predominantly in the counterclockwise (CCW) direction with occasional switches to clockwise (CW) (8). As long as all the filaments are spun CCW, they form a stable bundle. When one or more participating flagella switches to CW rotation, unbundling occurs, resulting in a so-called tumble event that likely brings a change in swimming direction once all the flagella resume CCW rotation (7). In a homogeneous environment, tumbles happen stochastically, whenever enough copies of the phosphorylated CheY protein (4) (CheY-P) diffuse to the motor and increase the chance of a CCW-CW switch through their interaction with the BFM (9,10). As a result, a single bacterium moves in the pattern of a random walk (11). The effective diffusion constant of such a random walk is linked to the tumble rate, usually considered a constant (12). The intracellular fraction of CheY that is phosphorylated is controlled by transmembrane proteins that act as chemosensors (4). They are able to bind very specific chemicals in the cell exterior and, in response, transiently increase or decrease the concentration of phosphorylated CheY-P inside the cell. This provides a mechanism for biasing the random walk by making tumbles more or less probable and ensuring, for example, that tumbles are less probable if the cell is moving toward a source of food. Transiently modifying its reorientation probability allows the bacterium to quickly respond to new changes in its surroundings and navigate gradients rather than just have a binary response to presence or absence of a chemical (13). This behavior, where the sensors modulate their own sensitivity to bring the CheY-P concentration to baseline levels only seconds after responding to a stimulus, is called perfect adaptation (14). It, however, works only if the successive stimuli are in approximately the micromolar range and thus are not over-saturating the sensors (15).

As changes in the rotational direction of single motors are at the root of this kind of directed motility in bacteria, the experimental quantity that is commonly used to express how often cells change direction is the CW bias, the fraction of time the motor spends rotating CW in a long enough time interval (10,14,16). As mentioned, in homogeneous environments, BFM switching events happen stochastically, and, since most studies on *E. coli*’s biased random walk have been done in dilute environments it is generally considered a fixed quantity (12). However, bacteria in nature have evolved, and typically live in complex environments such as the mammalian gastrointestinal tract or the soil. Consequently, interest is now shifting toward understanding how robust bacterial navigation is, when multiple competing stimuli are present, and when bacteria simultaneously swim and grow (5,17,18). For example, recent reports show higher steady-state CW bias in nutrient-rich environments compared with the commonly cited value found in dilute environments (12,18), lack of return of the CW bias to its pre-stimulus levels (19), and long-term increases in CW bias following shifts in osmolarities similar to values typical for the gastrointestinal tract (16). Because the biased random walk arises from transient changes in the CW bias due to the concentration gradient, and on the level of individual bacteria, changing the steady-state CW bias can affect the motion of the bacterial population in a complex manner.

Here, we therefore look to better characterize experimentally the changes in steady-state CW bias in different media, and subsequently explore, using a coarse-grained model of bacterial chemotaxis, the effects of these changes on the ability of bacteria to quickly and accurately find their target. The model describes the chemotactic trajectory within a concentration field as a chain of random steps. At each step, the state of the bacterium is represented in terms of its chemotactic behavior (either run or tumble) and its direction of movement, and updated problematically.

The biased random walk is not restricted to bacteria but is ubiquitous in the biosphere. Variations of it describe the movement patterns that arise when large herbivores search for new grazing patches (20) and the way *Drosophila* larvae search for optimal environmental temperatures (21). Our theoretical and experimental findings show that how it is biased becomes important. Thus, our results can be of wider relevance, e.g., for bio-inspired swarm robotics, either to design target search strategies (3) or as a means of controlling the spatial extent of swarms (22).

## Materials and methods

### *E. coli* growth and culturing

KAF84 cells were grown in tryptone broth (1% Bacto tryptone, 0.5% NaCl) at 30°C while being shaken at 200 rpm (1,10), supplemented with 100 μg mL^−1^ of ampicillin, and grown to optical density 600 (OD_600_) between 0.7 and 1.0 (Spectronic 200E Spectrophotometer; Thermo Scientific) (16). After growth, cells were washed in volume recovery buffer (VRB) composed of sodium motility buffer (NMB), which is a 10 mM sodium phosphate buffer, pH 7.1 (an aqueous solution with 6.1 mM Na_2_HPO_4_, 3.9 mM NaH_2_PO_4_ and 0.01 mM EDTA), with added glycine betaine, potassium chloride, and choline chloride to final concentrations of 10, 20, and 10 mM, respectively (23). These compounds are osmolytes needed for the cell to maintain its volume at higher osmolarities (24). NMB is a variant of the motility buffer, commonly used in flagellar motor and chemotaxis experiments (25), with sodium phosphates substituted for potassium phosphates. The latter is done to gain full control of potassium content in the buffer because potassium is also an osmolite (24). For experiments, cells were washed three times by centrifuging them into a pellet and replacing the supernatant with the buffer of choice. For experiments in VRB, all three washes were performed using VRB. For experiments with VRB + 200 mM sucrose and VRB + 400 mM sucrose, the final (third) wash was into those respective buffers. Cells were left to rest for approximately 15–70 min before microscopy (e.g., of the 237 recorded in VRB + 200 and VRB + 400 mM sucrose buffers, 77 were left to rest in the buffer between 15 and 30 min and 160 for 60–70 min), leaving enough time to dissipate any transient changes due to the sudden increase in extracellular osmolality (16) (for a summary of buffer compositions, see also Table 1).

**Table 1:** Table of buffers used in this work, together with their composition, osmolarity, and pH

Medium/buffer	Components	Osmolarity	pH
NMB	6.1 mM Na_2_HPO_4_ 3.9 mM NaH_2_PO_4_	24 mOsm/kg	7.12
VRB	NMB + 20 mM KCl 10 mM choline chloride 10 mM glycine betaine	92 mOsm/kg	7.04
MB0	NMB + 40 mM KCl 40 mM glycine betaine 40 mM NaCl	286 mOsm/kg	6.90
MB1	NMB + 80 mM KCl 80 mM glycine betaine 80 mM NaCl	501 mOsm/kg	6.87

All buffers are built on a sodium phosphate buffer, termed sodium motility buffer (see section “materials and methods”). To change the osmolarity of the buffers we used sucrose, as it is a sugar most laboratory *E. coli* strain do not metabolize (26). MB0, modified buffer 0; MB1, modified buffer 1.

### Microscopy and data collection

**Figure 1:**
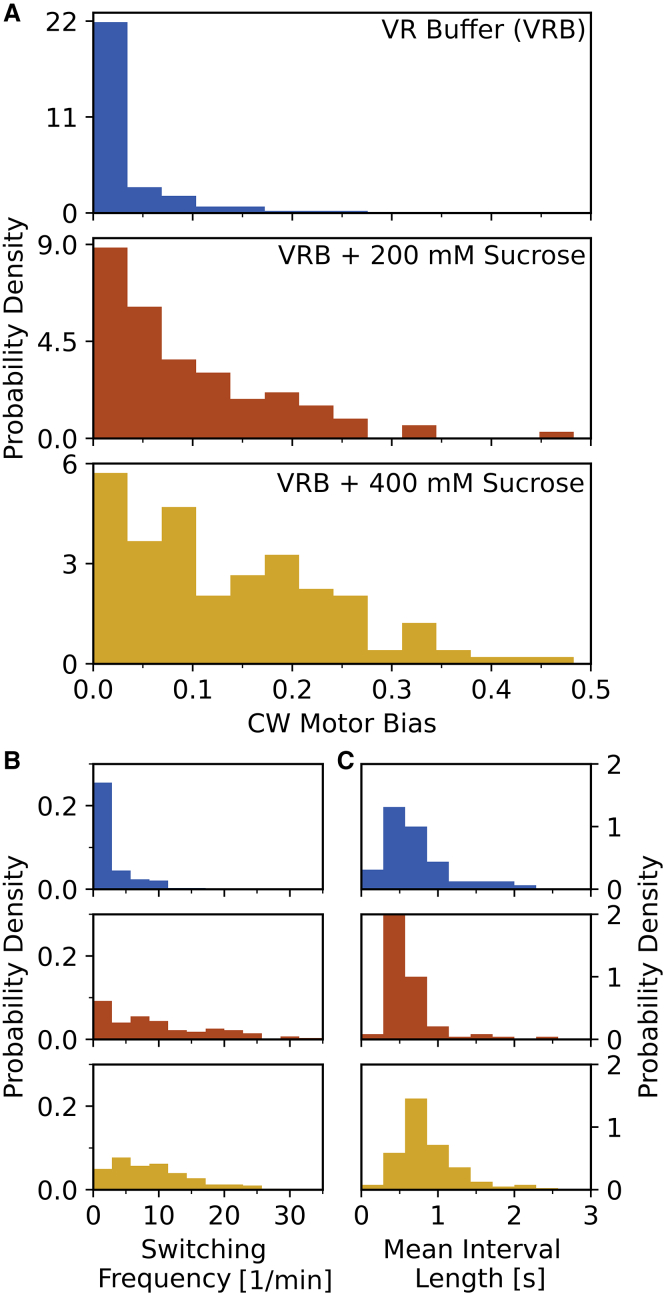
Variation in the parameters describing single bacterial flagellar motor rotational direction changes for the VRB buffer (top), VRB with addition of 200 mM sucrose (middle), and VRB supplemented with 400 mM sucrose (bottom). (*A*) Distributions of CW motor bias. (*B*) Distributions of the single motor CCW → CW switching frequency. (*C*) Distributions of mean single motor CW interval length. The VRB, VRB + 200 mM, and VRB + 400 mM sucrose conditions comprise 118, 95, and 142 single-cell, single-motor recordings respectively. See supporting material for experimental setup and Table 1 for buffer composition. To see this figure in color, go online.

As before, for BFM experiments, we used back-focal-plane interferometry (16,27,28). Briefly, the rotating bead is attached to a flagellar stub and placed into the focus of the heavily attenuated focused laser. The back-focal plane of the condenser is then imaged onto a position-sensitive detector (PSD Model 2931; New Focus). The voltage signal from the PSD was filtered and sampled as described before (16). The experiments were performed at room temperature (21°C ± 1°C). In Figs. 1, S1, and S2, we include 118, 95, and 23 cells recorded as part of our previous work (16) in the VRB buffer, VRB + 200 mM sucrose, and VRB + 400 mM sucrose conditions, respectively. In this work, we also expand the latter-most condition with 119 single-cell recordings, bringing the total number of motors assayed in VRB + 400 mM sucrose to 142. We record one motor trace per cell, so we refer to them as single-cell motor traces. Additionally, in Fig. S3 we included two CCW-biased motors observed in VRB buffer condition (these motors spend the majority of the time rotating in the CCW direction and occasionally switch to the CW direction). A small number of CCW-bias motors was observed before when cells were kept in 10 mM potassium phosphate buffer (12). The number of these is so small that it will not influence the population dynamics, so we have not included them in the CW bias distribution in Fig. 1. In Fig. S4 we included an additional 30 and 29 cells for the two new buffer conditions, respectively.

### Data analysis

**Figure 2:**
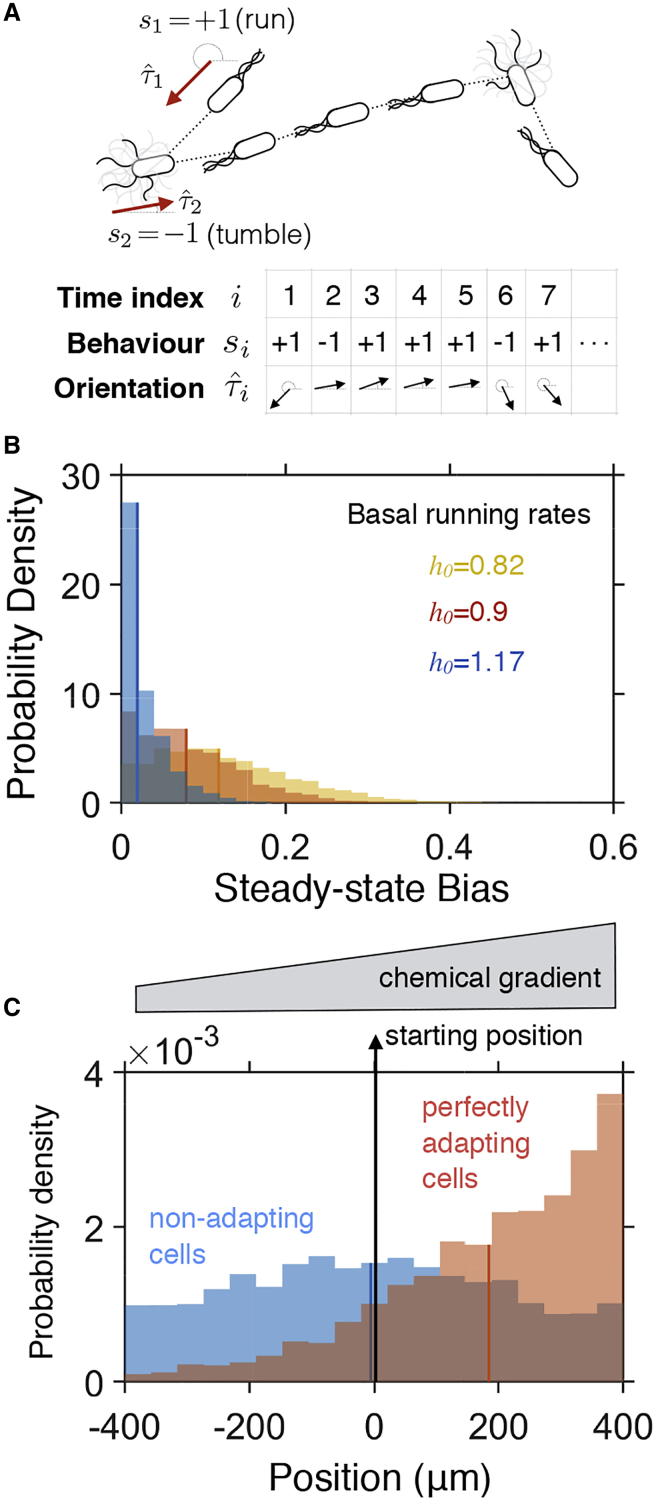
Persistent trajectory model of chemotaxis captures its basic features. (*A*) A chemotactic trajectory is modeled as a stochastic sequence of states, describing bacterial behavior (either s=+1 for ‘run’ or s=−1 for ‘tumble’) and direction of movement ($\hat{\tau }$). (*B*) Distribution of steady-state tumbling bias (fraction of time spent tumbling) for different values of the basal running rate, $h_{0}$, fitted to match the mean CW bias under different experimental conditions (see Fig. 1). Distributions were calculated from 10-s trajectories of $N=10^{5}$ cells. (*C*) Perfectly adapting cells (B=5) accumulate up a one-dimensional chemical gradient (0.01 AU⋅μm^−1^), whereas non-responding cells (B=0) are incapable of performing chemotaxis. Cell distributions were calculated from 20-s trajectories of N=5000 cells. Model parameters: Δt=0.1 s, v=20μm⋅s^−1^, ε=1, $k_{>}=1$, and $k_{<}=0.1$. To see this figure in color, go online.

X and Y signals obtained from voltages from the PSD were analyzed as before (16,23). Briefly, time-course traces were passed through a moving-window discrete Fourier transform to extract the motor speed as a function of time. These were then processed to calculate the CW bias, defined as the fraction of time the motor rotates CW in a given interval. Namely,

$$CWBias=\frac{N_{cw}}{N_{tot}},$$

where $N_{cw}$ is the number of data points corresponding to CW rotation and $N_{tot}$ is the total number of data points in a given time interval. For every single cell examined, 60 s of single-motor speed was taken as the interval for calculating CW bias (see also Fig. S2). The selection of the recording window maximizes our throughput and minimizes any photo damage (29) or sensitivity to slow fluctuations in CW bias (30). The single-cell motor switching frequency was calculated as the number of CCW → CW transitions per our 60-s interval (Fig. 2 *B*). In free-swimming cells, these transitions would most likely correspond to the initiation of a tumble event. CW → CCW events, corresponding to a resumption of a run, were not included in the count, and doing so would simply multiply the result by a factor of two. To obtain mean CW interval distributions, in Fig. 2 *C* we averaged the lengths of CW intervals from each single-cell motor trace in a given condition. These intervals serve as a proxy for tumble durations.

### Model simulation

To evaluate the path integrals (see also section “results”), we employ a particle filter (or sequential Monte Carlo) algorithm (31,32) to sample from the sequence of probability distribution $P\left(s_{1:t}\;{\hat{\tau }}_{1:t}|c\left(x\right)\right)\propto \;\text{exp}\;\left(-H\left(s_{1:t}\;{\hat{\tau }}_{1:t};c\left(x\right)\right)\right)$, t=2,…,T that describe the time evolution of a population of chemotactic agents in a concentration field c(x). The sampling algorithm proceeds as follows (see section “results” for definitions of parameters):

**Algorithm 1: Particle filter for sampling from the probability distribution sequence Pt≡Ps1:t,τˆ1:t|cx, t = 1, …,T**

**Input:** number of time-points, T; number of particles, N; concentration field, c(x); initial distribution, $\pi_{1}\left(s,\hat{\tau }\right)$, transition kernel, $M\left(s^{'},{\hat{\tau }}^{'}|s,\hat{\tau }\right)$.

**Output:** sample chemotactic trajectories $\left\{s_{1:T}^{i},{\hat{\tau }}_{1:T}^{\left(i\right)}\right\}$ for *i* = 1, …, *N*.

set t = 1

sample from initial distribution:

$\left(s_{t}^{\left(i\right)},{\hat{\tau }}_{t}^{\left(i\right)}\right)∼\pi_{1}$ for *i* = 1, …, *N*;

calculate weights:

$w_{t}^{\left(i\right)}=w_{t}\left(s_{t}^{\left(i\right)},{\hat{\tau }}_{t}^{\left(i\right)}\right)=1/N$ for *i* = 1,…*N*;

**for***t* = 2 ***to*** *T* **do**

 **for***t* = 1 ***to*** *N* **do**

 sample from previous (i.e., t − 1) population:

$a_{t-1}^{\left(i\right)}∼P\left(w_{t-1}^{\left(1\right)},\cdots ,w_{t-1}^{\left(M\right)}\right)$

 perturb state:

$\left(s_{t}^{\left(i\right)},{\hat{\tau }}_{t}^{\left(i\right)}\right)∼M\left(\cdot |s_{t}^{\left(a_{t-1}^{\left(i\right)}\right)},{\hat{\tau }}_{t}^{\left(a_{t-1}^{\left(i\right)}\right)}\right)$;

 calculate weight: $w_{t}^{\left(i\right)}=\frac{\exp\left(-H\left(s_{1:t},{\hat{\tau }}_{1:t};c\left(x\right)\right)+H\left(s_{1:t-1},{\hat{\tau }}_{1:t-1};c\left(x\right)\right)\right)}{M\left(s_{t}^{\left(i\right)},{\hat{\tau }}_{t}^{\left(i\right)}|s_{t}^{\left(a_{t-1}^{\left(i\right)}\right)},{\hat{\tau }}_{t}^{\left(a_{t-1}^{\left(i\right)}\right)}\right)}$;

 **end**

**end**

For the purpose of our numerical experiments, we set $\pi_{1}\left(s\text{,}\hat{\tau }\right)\propto \;\text{exp}\;\left(-h_{0}\frac{s+1}{2}\right)\cdot U\left(\hat{\tau }\right)$, and $M\left(s^{'}\text{,}{\hat{\tau }}^{'}|s,\hat{\tau }\right)=U\left(s^{'}\text{,}{\hat{\tau }}^{'}\right)$, where U(⋅) is the uniform distribution over all admissible states. A Matlab implementation of the algorithm can be found at https://git.exeter.ac.uk/mv286/chemotaxis-model.

## Results

### Steady-state CW bias changes in different media

We begin by experimentally characterizing CW bias in a range of different media. We know from previous work that step changes of some attractant/repellent concentrations can lead to new steady-state CW bias values in *E. coli* (33) and, similarly, that at higher osmolarities steady-state CW bias increases (16,23). While step changes in some attractant/repellent concentrations resulted in a change in CW bias (33), there was no obvious concentration dependency, but steady-state CW bias increased with the osmolarity for the previously measured three osmotic conditions (16). We, thus, focused on testing whether the increase in steady-state CW bias is an osmotic effect, as well as experimentally determining the steady-state CW bias distributions in different media. The latter will enable us to theoretically investigate the role of changes in the CW bias on chemotactic accumulation. The table of different buffers, their compositions, as well as the osmolarity we used are given in Table 1.

Fig. 1*A* shows CW bias mean value and distribution measured in three media differing only in osmolarity (16), which show a clear change. Next, in Fig. 1 *B* and *C*, we confirm that the change in CW bias is due to more frequent BFM switching events rather than longer periods spent rotating in the CW direction, and it is therefore relevant for the motion of bacteria. In Fig. S4, we perform the CW bias measurements in additional media different in composition as well as in osmolarity and conclude that osmolarity of the media plays a role in setting the CW bias but does not uniquely define it. A possible explanation for the role of osmolarity is the increased crowding in an already crowded intracellular environment (34). If osmotic pressure is kept constant at higher external osmolarities, for which there is experimental evidence (35), the concentration inside would be higher thus slowing down the diffusion of macro-molecules (36). Theoretical studies demonstrate that changes in macro-molecular diffusion can even slow down the growth rate (37). Changes in protein expression (up to sixfold), on the other hand, were shown to have little effect on the CW bias (38).

### Statistical mechanics description of bacterial chemotaxis gives description of whole chemotactic trajectories

Having characterized clear changes in the steady-state CW bias, we next theoretically study how these reflect on the speed and accuracy of finding the target. For the purpose, we propose a statistical mechanical description of chemotaxis of individual bacteria (Fig. 2). In contrast with previous models of chemotaxis that describe the stochastic phenomenology of CCW-CW switching and predict how cell density evolves over time (39, 40, 41, 42, 43, 44, 45), our model is built on a statistical description of chemotactic *trajectories*. In particular, single-cell trajectories are treated as stochastic sequences of runs and tumbles, allowing us to calculate probability distributions of any function of these trajectories as a path integral. This framework has mathematical similarities to wormlike chain (WLC) models describing semi-flexible biopolymers (46,47), and similar path-integral representations of bacterial chemotaxis have been used in the past to study chemotactic drift velocity (48,49).

The path integral is defined as the weighted sum over all possible individual trajectories of the bacteria; thus, the model naturally links the CW bias of individual bacteria with the behavior of the population. The underlying biochemistry is abstracted away but it is easy to generalize the model to incorporate more complex intracellular dynamics. The relevant length-scales in this framework are intermediate between those of PDE (partial differential equation) models (50), which capture average properties of populations but are insensitive to microscopic details, and mechanistic models that link behavior of individual bacteria to intracellular biochemistry (51, 52, 53, 54) but are more difficult to scale to experimentally realistic large populations. Furthermore, the path-integral approach is well suited to studying chemotaxis in complex, time-varying environments and could easily be extended to incorporate cell-to-cell interactions in space and time. Finally, it provides an efficient tool for the analysis of chemotaxis dynamics from time-lapse movies of swimming bacteria, or time-series recordings of motor rotational direction and speed.

In our model, we describe a chemotactic trajectory within a concentration field of chemoattractant/repellant, c(x), as a chain of random steps (indexed by *i*; Fig. 2 *A*). Each step *i* represents the state of the bacterium over a time window $\text{Δ}t_{i}=t_{i+1}-t_{i}$ in terms of 1) its chemotactic behavior $s_{i}$, either run ($s_{i}=+1$) or tumble ($s_{i}=-1$); and 2) its direction of movement, ${\hat{\tau }}_{i}$. The chemotactic signaling cascade is thus condensed and considered from the point of view of its final outcome. Furthermore, bacteria move only when in the run state at speed *v*. Hence, the bacterial position, $x_{i}$, follows from the chain description of the chemotactic trajectory and the initial position $x_{1}$:

$$x_{i}=x_{1}+v\overset{i-1}{\underset{j=1}{\sum }}{\hat{\tau }}_{j}\frac{\left(1+s_{j}\right)}{2}\Delta t.$$

State transitions, i→i+1, from $s_{i},{\hat{\tau }}_{i}$ to $s_{i+1},{\hat{\tau }}_{i+1}$ depend only the states, i,i+1. Hence, starting in state $x_{1}$, the probability of observing the sequence $\left(s_{1}\text{,}{\hat{\tau }}_{1}\text{,}s_{2}\text{,}{\hat{\tau }}_{2}\ldots s_{T}{\hat{\tau }}_{T}\right)\equiv \left\{s_{1:T}\text{,}{\hat{\tau }}_{1:T}\right\}$, where T≫1, is given by

$$P\left(\left\{s_{1:T}\text{,}{\hat{\tau }}_{1:T}\right\}|x_{1}\right)=p_{1}\prod_{i=1}^{T}p_{i\to i+1}\propto \;\text{exp}\left(-H\left(\left\{s_{1:T}\text{,}{\hat{\tau }}_{1:T}\right\}\right)\right)$$

with weight defined by:

$$H\left(\left\{s_{1:T}\text{,}{\hat{t}}_{1:T}\right\}\right)=\frac{\varepsilon }{2}\sum_{i=2}^{T}\left(1-s_{i}s_{i-1}\right)-\sum_{i=2}^{T}\frac{h_{i}}{2}\left(1+s_{i}\right)+\sum_{i=2}^{T}\kappa \left(s_{i-1}\right)\left(1-{\hat{\tau }}_{i}\cdot {\hat{\tau }}_{i-1}\right)$$

Increasing ε(>0) in the first term of *H* penalizes transitions between run and tumble states, noting that a typical run could extend for several steps. In the second term, $h_{i}$ controls the preference for running over the tumbling, which in general will depend on the exposure of the bacterium to the chemoattractant/repellent as it moves through the concentration profile, c(x). Here, we enforce perfect adaptation by making $h_{i}$ depend linearly on the concentration gradient; i.e., $h_{i}=h_{0}+B{\hat{\tau }}_{i}\cdot \nabla c\left(x_{i}\right)$. Parameter $h_{0}$, hereafter referred to as the basal running rate, controls the distribution of steady-state tumbling bias (fraction of time a bacterium spends tumbling) (Fig. 2 *B*). Furthermore, parameter *B* controls the strength of the chemotactic response to the chemical gradient, with B=0 indicating non-responding cells and B>0 (B<0) perfectly adapting cells to chemoattractant (chemorepellent) concentrations (Fig. 2 *C*). Thus, ε, $h_{i}$, and *B* represent the tuning of the bacterial swimming that is a result of the action of the entire chemotactic network, without considering the detailed protein interactions. For example, to achieve perfect adaptation, chemotactic networks ensure an efficient dephosporylation of CheY-P shortly after it has been phosporylated. The effect is that the cell is ready to sense and respond to a new changes in the environment. Here we achieve the same by making the $h_{i}$ dependent on the position in the gradient. For simplicity, and without the loss of generalization, we assume every change in rotational direction of a motor results in a tumble; hence, hereafter we use the terms CW bias and tumbling bias interchangeably. The model could be extended to include any mathematical relation between a cell’s run/tumble bias and the number and CW bias of the motors, such as the one experimentally observed previously (55). Finally, the third term of *H* controls the change of orientation between steps, which depends on the chemotactic state. Since reorientation is significantly larger during tumbling,

$$\kappa \left(s_{i}\right)=\left\{ \begin{array}{ll} k_{>} & s_{i}=-1\\ k_{<} & s_{i}=+1 \end{array}\right.$$

with $k_{>}\gg k_{<}$. The final bacterial position is given by $x\left(t_{T}\right)=x_{j},j=T$.

Setting ε=h=0 and κ constant reduces this model to the classic wormlike chain of polymer physics. We evaluate the path integral numerically (see also section “materials and methods”), using a constant time step equal to the duration of a typical tumble event; i.e., Δt=0.1 s (11,56); constant speed corresponding to the average running speed on glucose (i.e., v=20μm⋅ s^−1^ (1,11)); and ε=1, B=5, $k_{>}=1$, $k_{<}=0.1$.

**Figure 3:**
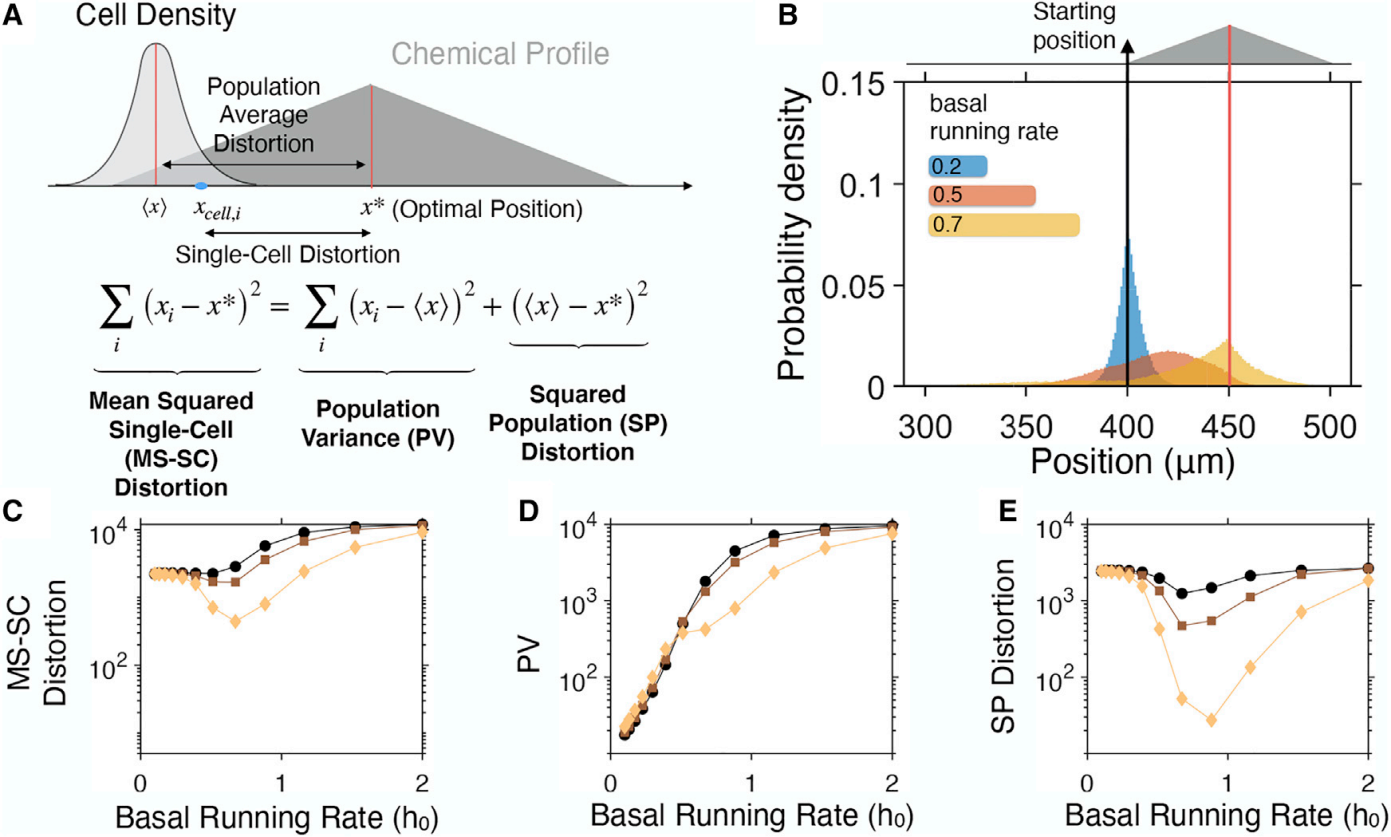
Bacterial chemotaxis speed and accuracy are influenced by the basal running rate. (*A*) Schematic illustration of a triangular chemotactic profile in one-dimensional space. Chemotactic bacteria will seek to move toward the optimal position ($x^{\text{∗}}$). We use the term distortion to denote the distance of single bacteria to $x^{\text{∗}}$, and population-average distortion for the distance between the population-average position and $x^{\text{∗}}$. MS-SC distortion, mean squared single-cell distortion; PV, population variance; SP, squared population distortion. (*B*) Accumulation of bacterial populations with different basal running rates in a triangular chemical profile. Each population consists of $N=10^{6}$ cells, initialized at the left base point of the triangular profile and followed over 10 s. (*C*) Mean squared single-cell distortion, (*D*) PV, and (*E*) squared population distortion as a function of the basal running rate for different heights of the triangular profile. Markers correspond to different gradients of the triangular profile; i.e., 0.005 (○), 0.01 (□), and 0.02 (◇) AU⋅μm^−1^. Model parameters: Δt=0.1 s, B=5, v=20μm⋅ s^−1^, ε=1, $k_{>}=1$, and $k_{<}=0.1$. To see this figure in color, go online.

We next select the environment, by taking into account that, if we wish to characterize how well chemotaxis “maps” the environment to find (un)favourable regions, we need a profile with more structure than a concentration gradient. We select a triangular-shaped profile in one-dimensional space (Fig. 3 *A*) and for it define “perfect chemotaxis” as all the bacteria going to the peak (and staying there). While achieving this is impossible, we can study how close the bacteria can get to this situation given different steady-state tumbling bias values. For the purpose, we follow the chemotactic response of bacterial populations initially positioned at the tip of the base of the triangular profile (Fig. S5), and each with a different basal running rate ($h_{0}$). Fig. 3 *B* illustrates how the basal running rate modulates the speed and accuracy with which cells find the target. Lower values of $h_{0}$ (Fig. 3 *B*; blue population) achieve consistent exploration of the chemical profile and hence less cell-to-cell variability. However, this comes at a cost of a slower average movement of the bacterial population toward the target. As $h_{0}$ is increased, a fraction of the cells approach the target, but the dispersion of the population increases, with a portion of cells completely missing the chemoattractant-rich area (left tail of the red population in Fig. 3 *B*). Higher values of $h_{0}$ give rise to higher levels of heterogeneity, as prolonged running periods enable cells to disperse faster and miss the target (Fig. 3 *B*, yellow population).

To quantify these observations, we introduce the mean squared single-cell (MS-SC) distortion, which is the mean squared distance of a single-cell position, $x_{i}$, from the optimal position; i.e., the peak of the triangular profile, $x^{\text{∗}}$ (see Fig. 3 *A*). The MS-SC distortion can be decomposed into a sum of two terms: 1) the population variance (PV; proxy for positional entropy), and 2) the squared population distortion (SP distortion; proxy for aggregate chemotactic effectiveness):

$$\underset{\text{MS}-\text{SC}}{\underset{︸}{\left\langle {\left(x_{i}-x^{\text{∗}}\right)}^{2}\right\rangle }}=\underset{\text{PV}}{\underset{︸}{\left\langle {\left(x_{i}-\left\langle x\right\rangle \right)}^{2}\right\rangle }}+\underset{\text{SP}}{\underset{︸}{{\left(\left\langle x\right\rangle -x^{\text{∗}}\right)}^{2}}}$$

where $\left\langle \cdot \right\rangle =\frac{1}{N}\sum_{i=1}^{N}\cdot$ denotes averaging over the bacterial population. We note that the MS-SC distortion decreases as the population becomes concentrated around the optimal position and becomes zero in the case of “perfect chemotaxis.” The equation also highlights that chemotactic strategies that achieve the same SP distortion levels can demonstrate varying MS-SC distortion levels and vice versa.

Fig. 3*C*–*E* shows the three terms in the equation (MS-SC distortion, PV, and SP distortion) as a function of the basal running rate ($h_{0}$), quantifying the connection between response accuracy and population heterogeneity. For example, when faced with shallow gradients (Fig. 3 *C*–*E*, black circles), single cells suffer on average from higher distortion as we increase the basal running rate (Fig. 3 *C*). The effect could go unobserved if we focus only on the mean of the population coming closer to the target (Fig. 3 *E*), disregarding the fact that, at the same time, the variability in the population is increasing rapidly (Fig. 3 *D*). Furthermore, for the range of gradients we examined, the SP distortion demonstrates a non-monotonic behavior as a function of $h_{0}$. This optimal value suggests that the basal running rate is another chemotactic variable that bacterial populations could use to adapt to different environmental conditions. This adaptation process could be direct, via regulation of intracellular components, or indirect, as cells with lower distortion levels will have a growth advantage. Interestingly, the values of $h_{0}$ inferred from the CW bias data (0.82–1.17; see Fig. 2) are around the values achieving minimum distortion levels in Fig. 3.

**Figure 4:**
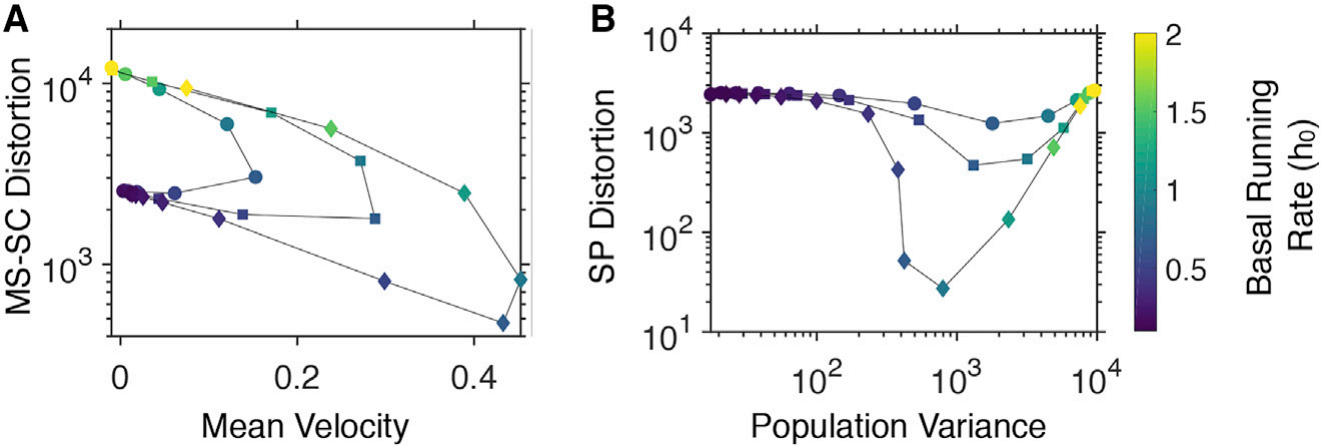
Trade-off on chemotactic speed and accuracy imposed by different running rates. (*A*) Mean squared single-cell distortion versus mean velocity of a bacterial population in a triangular profile as the basal running rate (color coded) is varied. Optimal basal running rate achieves the highest mean velocity and lowest distortion. Markers correspond to different gradients of the triangular profile (0.005 (○), 0.01 (□), and 0.02 (◇) AU⋅μm^−1^). (*B*) Squared population distortion versus populational variance of a bacterial population in a triangular profile at different basal running rate (color coded). The optimal basal running rate achieves the lowest distortion and positional variance simultaneously. Different lines correspond to different heights of the triangular profile. To see this figure in color, go online.

Fig. 4*A* illustrates that basal running rate can be controlled to maximize chemotactic speed and accuracy. Low $h_{0}$ gives rise to low population velocity due to the increased times spent in the tumble state. High $h_{0}$, on the other hand, enables cells to run for longer, but obstructs them from integrating adequate information about the chemoattractant concentration. The latter gives rise to higher MS-SC distortion as well as lower average velocity. For intermediate values of $h_{0}$, the system demonstrates optimal levels of velocity and accuracy. This finding highlights the system’s two competing requirements (57): fast response to environmental changes (leading to high velocity) versus robust longer-term accumulation at chemoattractant peaks (i.e, low MS-SC distortion). Similarly, Fig. 4 *B* illustrates that successful chemotactic strategies for the entire bacterial population involve intermediate values of $h_{0}$, where the PV remains low as the population mean comes close to the target (low population distortion).

**Figure 5:**
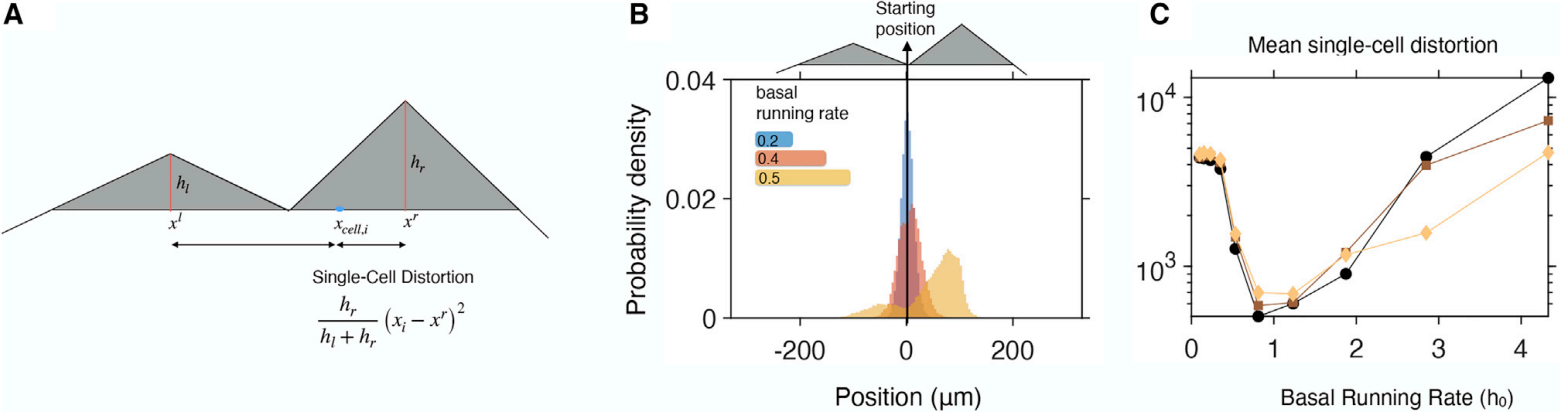
Chemotactic efficiency in a double-peak chemical profile. (*A*) Schematic illustration of a chemical profile in one dimension consisting of two peaks. To quantify the chemotactic accuracy at the single-cell level, we define the single-cell distortion as the squared distance to the closest peak weighted by the relative peak height. (*B*) Accumulation profiles of bacterial populations with different basal running rates. Each population consists of $N=10^{5}$ cells, initialized between the two peaks and followed over 30 s. (*B*) Mean single-cell distortion as a function of the basal running rate. Markers correspond to different heights (AU⋅μm^−1^) of the two triangles: ○ 0.005 (left) and 0.01 (right); □ 0.0025 (left) and 0.01 (right); ◇ 0.00125 (left) and 0.01 (right). Model parameters: Δt=0.1 s, B=5, v=20μ m⋅s^−1^, ε=1, $k_{>}=1$, and $k_{<}=0.1$, $x^{l}$ = −100 μm, $x^{r}$ = 100 μm. To see this figure in color, go online.

To further test our theoretical result, which states $h_{0}$ is another chemotactic variable that can be optimized, we consider a more complex chemical profile. Specifically, two peaks in one dimension with the bacterial population initially positioned between them (Fig. 5 *A*). Here, we quantify the chemotactic efficiency of individual bacteria by calculating the single-cell distortion ($D_{\text{s}c}$), which is defined as the squared distance to the closest peak ($x^{\text{∗}}$) weighted by the relative peak height:

$$D_{\text{s}c}=\frac{h^{\text{∗}}}{h_{l}+h_{r}}{\left(x_{i}-x^{\text{∗}}\right)}^{2}$$

where $x_{i}$ is the position of the cell; $x^{\left(\cdot \right)}$, $h^{\left(\cdot \right)}$ is the position and the height of each peak (l or r); and $x^{\text{∗}}$, $h^{\text{∗}}$ correspond to the position and height of the peak closest to the $x_{i}$. Fig. 5 *B* illustrates how the bacterial population disperses in space for different basal running rates, and once again the basal running rate controls the speed and accuracy of bacterial chemotaxis: low values of $h_{0}$ result in slow exploration of the chemical profile, while high values of $h_{0}$ result in faster but less accurate search. Hence, we observe that intermediate basal running rates present an optimum strategy allowing cells to reach closer to the peak in a time-efficient manner (Fig. 5 *B*). We note that precise optimum value of $h_{0}$ will depend on the relevant time scale, since, at longer timescales, lower $h_{0}$ strategies enable a more thorough search of the space.

Finally, we note that, similarly to experimentally observed changes in CW bias (i.e., changes in basal running rate), bacterial running speed can also change, either metabolically or in response to attractants (1,58), which can affect the speed and accuracy of bacterial accumulation and must be taken in account to optimize the chemotactic response in complex environments (see supporting material and Fig. S6).

## Discussion

Our experimental results highlight that studying bacterial motility in environments closer to their natural habitat can uncover adaptations, which can be relevant for their accumulation, as suggested by our model results. Our model also provides a novel, parsimonious description of bacterial chemotaxis at the single-cell level, capturing all the key features of its phenomenology. The statistical character of the model provides access not only to single-cell chemotactic dynamics as other agent-based chemotaxis models do (54,59) but also allows computationally efficient estimation of population measures, bridging the gap between the two scales of description. Despite its simplicity, the model can be straightforwardly extended to capture more realistic modes of chemtotaxis (for example, in two- or three-dimensional space, or accounting for changes in the running speed of cells and responses that are not perfectly adapting), and study how such modes affect bacterial accumulation. With advancements in observational techniques and manipulation methods used to probe bacterial chemotaxis in complex environments, our model could provide an efficient inference tool for identifying tumble/run events and characterizing single-cell chemotactic responses in more realistic scenarios. Finally, the influence of the reorientation frequency of individuals within a population on the population level speed and accuracy of reaching a target could inspire search algorithms used in unmanned aerial vehicles (60,61).

## Author contributions

M.V., J.R., T.P., and T.B.L. designed the research, developed the model, and analyzed the data. J.R. carried out experiments. M.V. contributed model simulation methods. M.V., J.R., T.P., and T.B.L. wrote the paper.

## Supporting material

Document S1. Figures S1–S6

Document S2. Article plus supporting material
