# Supplementary material for: Steady-state running rate sets the speed and accuracy of accumulation of swimming bacteria
Source: Biophys J. 2022 Aug 31;121(18):3435–44. doi: 10.1016/j.bpj.2022.08.012 (PMC9515231; doi:10.1016/j.bpj.2022.08.012)
Supplement: Document S1. Figures S1–S6 [file mmc1.pdf]

Biophysical Journal, Volume *121*

Supplemental information

Steady-state running rate sets the speed and accuracy of accumulation of swimming bacteria

Margaritis Voliotis, Jerko Rosko, Teuta Pilizota, and Tanniemola B. Liverpool

Supplementary Information: Steady state running rate sets the speed and accuracy of accumulation of swimming bacteria

M. Voliotis, J. Rosko, T. Pilizota, T. B., Liverpool

June 14, 2022

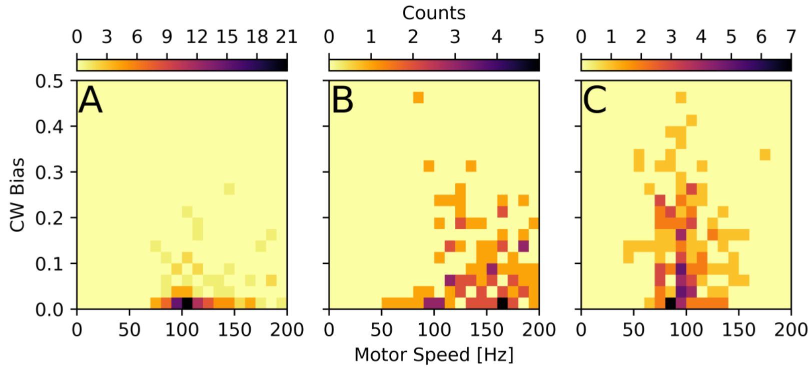

Figure S1: 2D histograms showing lack of dependency between the CW Bias and the speed of flagellar motors in the experimental conditions presented in the main text Fig. 1. The CW Bias is presented on the Y-axis, and the motor speed on the X. The color map, above each panel, represents the number of motors having a rotational speed and CW Bias in the bin defined around a given speed and CW Bias. The bin widths are 10 Hz for speed and 0.025 for CW Bias. Speeds were extracted from single cell measurements as described in *Data Analysis* and the value presented here is the average CCW (run) speed over a 60 s interval. CW Bias was calculated using the same interval, using Eq. 1 in the main text. The panels (A), (B), and (C) represent the "VRB", "VRB+200 mM sucrose" and the "VRB + 400 mM sucrose" experimental conditions, respectively. The number of single cells analysed in each of these conditions is 118, 95 and 142.

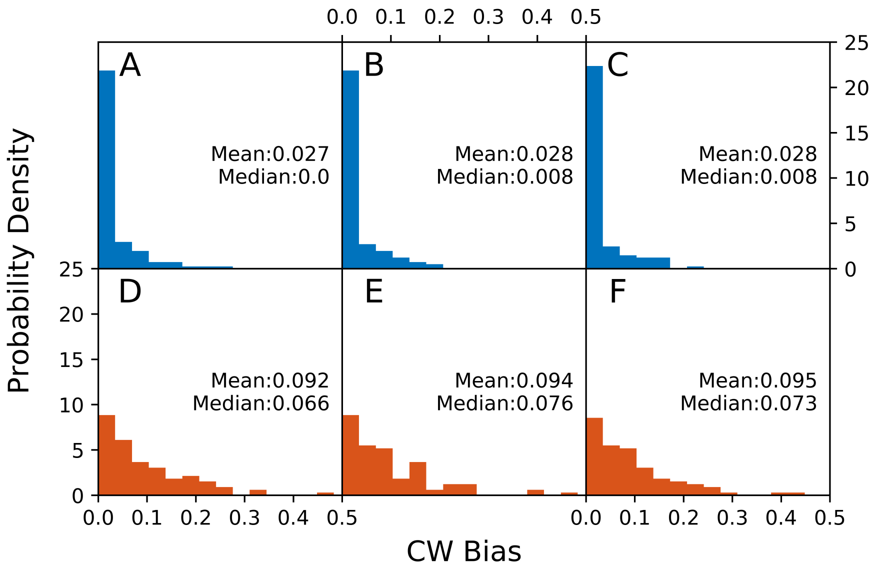

Figure S2: Histograms showing that the mean CW Bias and the shape of the distribution do not significantly change if the sampling interval, described under *Methods* in the main text, is increased from 60 s to 120 or 180 s. Panels (A-C) contain the histograms for VR Buffer (VRB), constructed from single cell/single motor recordings lasting 60, 120 or 180 s, respectively. Bin width is 0.033. Panels (D-F) are analogous but for the VRB + 200 mM sucrose condition.

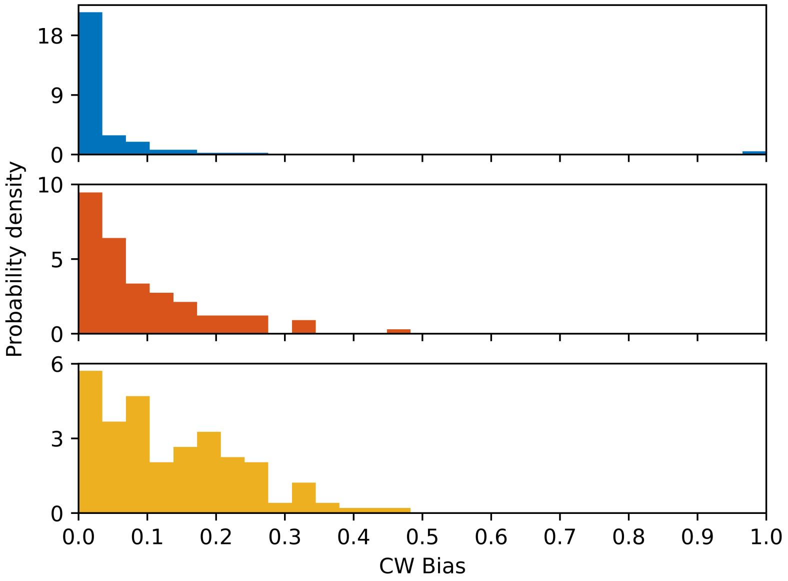

Figure S3: CW Bias distribution from the main text Figure 1, reproduced with inclusion of two CW Biased motors in the 'VRB' Buffer condition. The conditions (top to bottom) are VRB Buffer (Blue), VRB with addition of 200 mM sucrose (Orange) and VRB supplemented with 400 mM sucrose (Yellow).The VRB, VRB+200 mM sucrose and VRB+400 mM sucrose conditions comprise 120, 95 and 142 single cell, single motor recordings respectively. Buffer compositions are given in Table 1 in the main text.

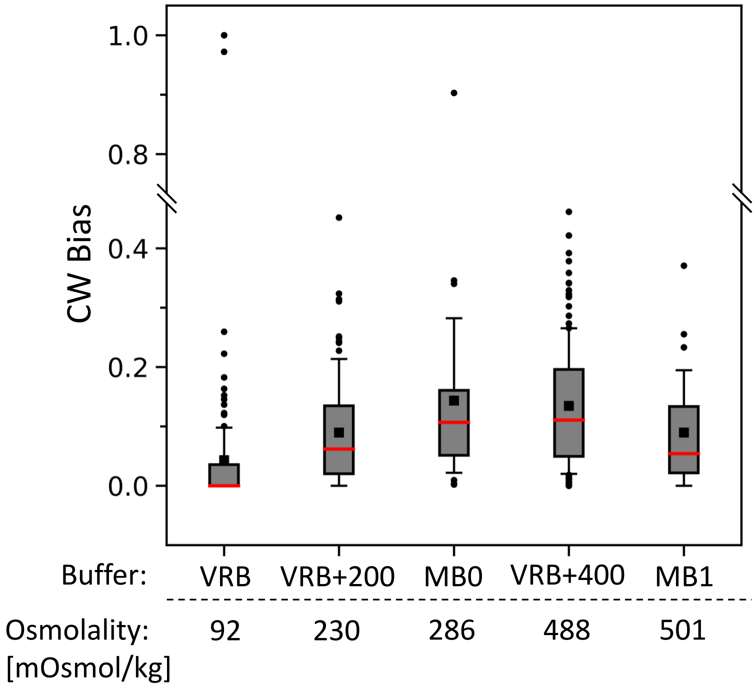

Figure S4: Box plots of CW Motor Biases for buffers from the main text, 'VRB', 'VRB + 200 mM sucrose', and 'VRB + 400 mM sucrose', as well additional two buffers, termed MB0 and MB1. The plots are in order of increasing buffer osmolality, and contain 120, 95, 30, 142 and 29 single cell measurements, respectivelly. For more information of buffer compositions consult Table 1 and *Methods* in the main text.

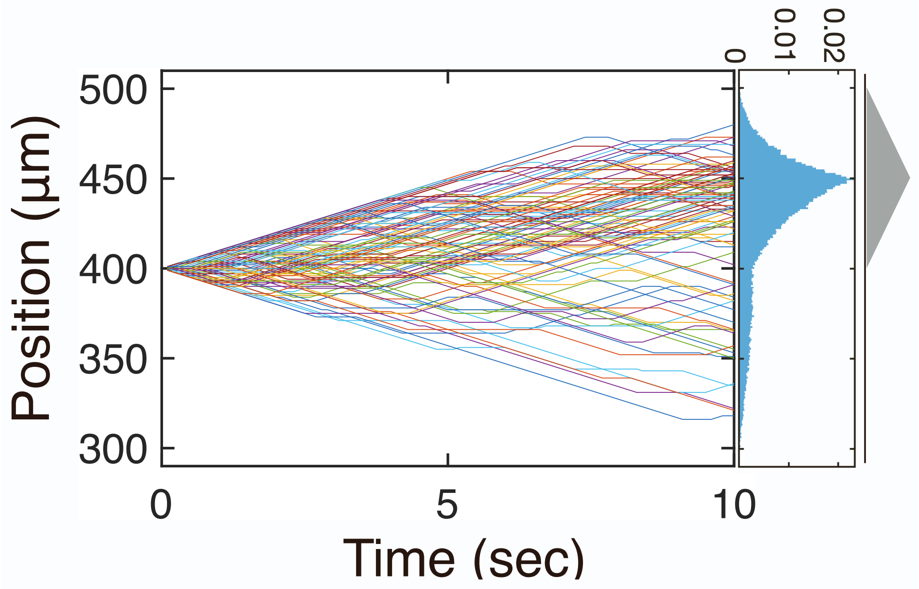

Figure S5: Sample bacterial trajectories in a triangular chemical profile. (Right) Accumulation of a bacterial population ($N = 10^6$ cells) after 10 s and schematic illustration of the chemical profile. The gradient of the triangular chemical profile is 0.01 AU$\cdot\mu$m^{-1}
.

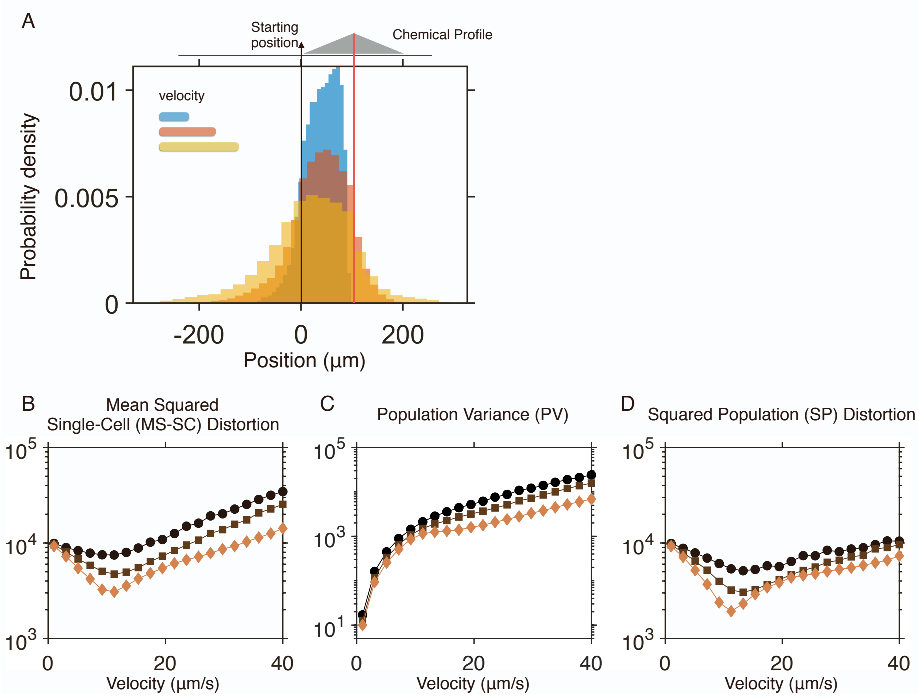

Figure S6: Effect of swimming velocity on the accumulation of bacterial populations. (A) Accumulation of bacterial populations with different velocities (10, 20, and 30 μms^{-1}) in a triangular chemical profile (gradient 0.01 AU$\cdot\mu$m^{-1}). Each population consists of $N = 5 \cdot 10^5$ cells, initialised at the left base point of the triangular profile and followed over 10 s. (B) Mean squared single-cell distortion, (C) population variance and (D) squared population distortion as a function of the basal running rate for different heights of the triangular profile. Different markers correspond to different gradients of the triangular profile, i.e., 0.005 (◯), 0.01 (□), and 0.02 (◇) AU$\cdot\mu$m^{-1}.
